# Population Size and the Rate of Language Evolution: A Test Across Indo-European, Austronesian, and Bantu Languages

**DOI:** 10.3389/fpsyg.2018.00576

**Published:** 2018-04-27

**Authors:** Simon J. Greenhill, Xia Hua, Caela F. Welsh, Hilde Schneemann, Lindell Bromham

**Affiliations:** ^1^ARC Centre of Excellence for the Dynamics of Language, Australian National University, Canberra, ACT, Australia; ^2^Department of Linguistic and Cultural Evolution, Max Planck Institute for the Science of Human History (MPG), Jena, Germany; ^3^Research School of Biology, Macroevolution and Macroecology, Australian National University, Canberra, ACT, Australia

**Keywords:** language evolution, language phylogenies, computational historical linguistics, demography, population size, Galton's problem, phylogenetic independence

## Abstract

What role does speaker population size play in shaping rates of language evolution? There has been little consensus on the expected relationship between rates and patterns of language change and speaker population size, with some predicting faster rates of change in smaller populations, and others expecting greater change in larger populations. The growth of comparative databases has allowed population size effects to be investigated across a wide range of language groups, with mixed results. One recent study of a group of Polynesian languages revealed greater rates of word gain in larger populations and greater rates of word loss in smaller populations. However, that test was restricted to 20 closely related languages from small Oceanic islands. Here, we test if this pattern is a general feature of language evolution across a larger and more diverse sample of languages from both continental and island populations. We analyzed comparative language data for 153 pairs of closely-related sister languages from three of the world's largest language families: Austronesian, Indo-European, and Niger-Congo. We find some evidence that rates of word loss are significantly greater in smaller languages for the Indo-European comparisons, but we find no significant patterns in the other two language families. These results suggest either that the influence of population size on rates and patterns of language evolution is not universal, or that it is sufficiently weak that it may be overwhelmed by other influences in some cases. Further investigation, for a greater number of language comparisons and a wider range of language features, may determine which of these explanations holds true.

## Introduction

The role of speaker population size in shaping patterns and rates of language and cultural evolution has been much discussed, but few generalities have been agreed upon. It has been suggested that larger populations should have higher rates of language change, because populations containing more individuals provide more opportunity for innovations to arise (Richerson et al., 2009; Kline and Boyd, 2010; Baldini, 2015). Large populations might also be less prone to random sampling effects that can cause elements of language and culture to be lost (Shennan, 2001; Henrich, 2004; Kline and Boyd, 2010; Collard et al., 2013) and they may have less stringent norm enforcement allowing them to change faster (Bowern, 2010; Trudgill, 2011). Larger populations might also have more robust transmission systems: having more people to learn from might increase fidelity of information transition (Derex et al., 2013), possibly because learners in large populations have a large set of potential models to learn from (Henrich, 2004; Kline and Boyd, 2010). Exposure to more people may make learning more robust, potentially allowing retention of a wider range of linguistic diversity (Trudgill, 2004; Hay and Bauer, 2007; Atkinson, 2011; Wichmann et al., 2011; Derex et al., 2013), although this effect is not universally supported (Caldwell and Millen, 2010; Read, 2012).

Other researchers have proposed that rates of change should be fastest in small populations due to the more rapid diffusion of new features (Nettle, 1999). Languages spoken by small speaker populations might be able to develop and retain greater linguistic complexity (Nettle, 2012). Smaller populations may have greater tolerance of diversity (Milroy and Milroy, 1985, 1992) and more malleable linguistic representations (Lev-Ari, 2017) which could speed up rates of change. Further, it has been suggested that the rate of language change may be accelerated by serial founder effects as new languages are started from relative small populations (Atkinson et al., 2008), which could increase the rate of loss of language elements from the ancestral language (Trudgill, 2004; Atkinson, 2011). Small speaker populations may also be more influenced by language contact through trade and marriage across groups, which might increase rates of language change (Bowern, 2010).

In contrast, other studies have found little or no significant effect of population size on the rate of language change or phoneme inventory size (Wichmann and Holman, 2009; Moran et al., 2012). If languages evolve in a purely stochastic manner, analogous to neutral molecular evolution, then rates of change might be independent of population size (Neiman, 1995; Shennan and Wilkinson, 2001; Bentley et al., 2004). The controversial claim that the average rate of word turnover is essentially the same in all languages, has led to much-disputed attempts to date language diversification by assuming a uniform rate of change over time (for examples of contributions to this debate see: Swadesh, 1952, 1955; Hoijer, 1956; Rea, 1958; Bergsland and Vogt, 1962; Sankoff, 1970; Blust, 2000). A similar effect has been suggested for cultural evolution because, for a variety of cultural traits from Neolithic pottery motifs to modern American pop songs, the frequency of variants matches the predictions of a purely stochastic model such that the rate of change is reasonably regular (Bentley et al., 2007).

So, despite many studies on a wide range of languages and language features, there is no consensus on whether population size has a consistent influence on patterns and rates of linguistic evolution (Bowern, 2010; Greenhill, 2014). The lack of a consistently predictable influence of population size on language change might indicate that it is not a universally important factor in rates of language change. Alternatively, the inconsistent patterns might also be due to complicated patterns of change. For example, if rates of word gain show different relationships with population size than rates of word loss, then overall rates of change may show no consistent pattern, and the patterns uncovered in any study might depend on the mode of measuring language change (Bromham et al., 2015a). The diversity of conclusions in published studies could also arise from the diversity of languages studied, data types analyzed, or methodological approaches.

Testing these hypotheses has been challenging for several reasons. Most studies analyzing rates of language change have focused on features within one language (e.g., Johnson, 1976), or relied on simulations (e.g., Nettle, 1999), making it difficult to draw general conclusions about language change. Comparative studies of language change also need a way of overcoming the problem of statistical non-independence due to relatedness. Since languages evolve and diversify from shared ancestors, closely related languages are likely to be more similar to each other in many ways. This similarity by descent means that any association between the two traits might simply be due to the co-occurrence of the traits in a common ancestor, even if there is no functional connection between the two. Therefore, statistical tests cannot treat each language as an independent piece of evidence about the relationship between population size and the patterns of language evolution. This methodological problem, often referred to as *Galton's problem*, can confound attempts to find relationships between language and demographic factors (Moran et al., 2012; Roberts and Winters, 2013).

Our aim in this paper is to examine the influence of one aspect of demography (size of speaker population) on one aspect of language evolution (the gain and loss of words from basic vocabulary). Specifically, we wish to test whether the association between population size and rates of word gain and loss noted in a study of 10 pairs of Polynesian languages reflects a general pattern. The study of Polynesian languages compared the gain and loss of cognate terms for basic vocabulary and demonstrated greater rates of word gain in larger populations and greater rates of word loss in smaller populations (Bromham et al., 2015a). In many ways, Polynesia represents a perfect “laboratory” of language evolution, with a recent, well-characterized history of colonization of previously uninhabited islands (Goodenough, 1957). Most Polynesian languages are restricted to clearly-defined groups of islands, and the population size of speakers is closely correlated with the area inhabited (Bromham et al., 2015a). As they are the product of a recent human expansion (Spriggs, 2010), Polynesian cultures, and languages share many similarities (Pawley, 1967) and are largely found in similar environments (Kirch and Green, 1987). While these features make Polynesia an ideal case study in language evolution, it also makes it difficult to extrapolate from the patterns observed in Polynesia to general patterns of language evolution. Do languages spoken in other parts of the world by much larger groups of people with wider continental distributions show similar patterns?

**Figure 1:**
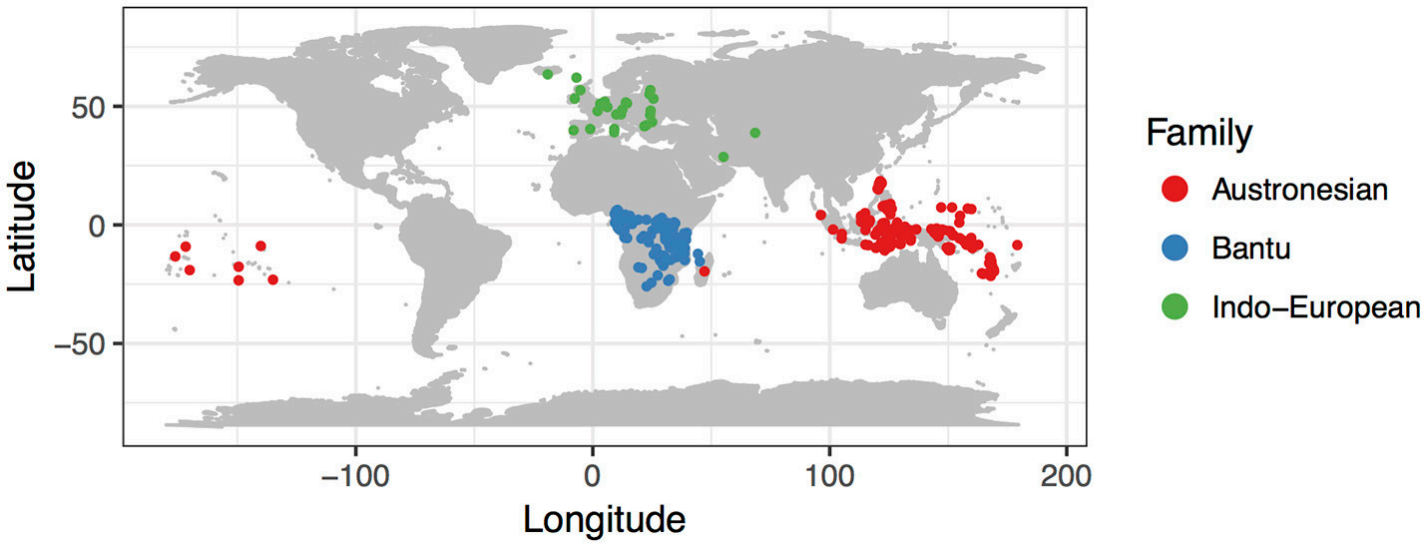
Map of languages included in this study. Each point represents the mid-point of the area occupied by one of the languages included in our study (see Tables 1–3).

To test the generality of the relationship between population size and rates of word gain and loss, we chose 153 pairs of closely related sister languages from three of the largest language families, Austronesian, Indo-European, and Niger-Congo (Bantu subfamily). The languages in our analysis are from a wide geographic area, from the North Atlantic to the South Pacific (Figure 1). These language pairs span a huge range of speaker population sizes, from Perai to Aputai spoken on the island of Wetar in the Maluku province of Indonesia (spoken by 280 and 150 people, respectively), to Sambaa and Bondei spoken in the mountain regions of Northern Tanzania (664,000 and 50,000 people), to German and Luxembourgish in continental Europe (spoken by 69,800,000^1^ and 266,000 people respectively). For each of these families, we used published linguistic databases of basic vocabulary to evaluate relative rates of word gain and loss, using a technique that explicitly accounts for non-independence due to the relatedness of the languages.

## Materials and methods

### Language families

We analyzed data from three of the largest language families, Austronesian, Indo-European, and Niger-Congo (Bantu subfamily). These language groups span a large range of population sizes, a wide geographic area and varied cultures and histories, which allows us to test the generality of the influence of population size on rates of language change (Figure 1).

The Austronesian language family is the world's second largest, containing 1,274 languages spoken across a wide range of islands as well as on continental landmasses, from Madagascar to Southeast Asia and the Pacific (Hammarström et al., 2016). There are 10 major Austronesian sub-groups, nine of which contain only 20 languages in total, and are spoken by indigenous Formosan people in Taiwan (Blust, 2013). The other languages form the Malayo-Polynesian group, which began diversifying around 4,000 to 4,500 years ago in a series of expansions across the Pacific Ocean (Gray et al., 2009; Hung et al., 2011; Spriggs, 2011; Amano et al., 2013; Ko et al., 2014; Blust, 2015). Austronesian societies include hunter-gatherer groups (e.g., the Mikea in Madagascar), agriculturalists (e.g., the Saisiyat in Taiwan), and complex socially-stratified societies such as in Java or Bali (Geertz, 1959; Jay, 1969). Austronesian languages vary greatly in their range and degree of isolation (Gavin and Sibanda, 2012), from remote Pacific islands containing a single indigenous language, to the diverse larger islands and landmasses of Southeast Asia and Near Oceania where many different languages may come into contact.

The Indo-European language family contains 581 languages in 8–10 sub-families, including many of the languages of Europe (e.g., English, Spanish, Portuguese, Russian), as well as many spoken in the Middle East and India (e.g., Bengali, Farsi, Hindi, Punjabi). The origin of the family is debated: while some place the origin in the Russian Steppes 5,000 years ago (Anthony and Ringe, 2015; Chang et al., 2015; Haak et al., 2015), others date it to Anatolia 8,000 years ago (Renfrew, 1987; Gray and Atkinson, 2003; Gray et al., 2011; Bouckaert et al., 2012). However, the uncertainty concerning the origin of the family does not affect our analysis of closely related sister pairs.

The Niger-Congo languages comprise the world's largest language family with 1,430 languages spoken across sub-Saharan Africa (Hammarström et al., 2016). The Bantu languages (550 languages), one of the major subgroups of Niger-Congo, are thought to have originated between 4,000 and 5,000 years ago in west central Africa, perhaps near the Nigerian-Cameroon border, and expanded south through the rainforest (Berniell-Lee et al., 2009; Montano et al., 2011; Pakendorf et al., 2011; de Filippo et al., 2012; Currie et al., 2013; Li et al., 2014; Grollemund et al., 2015).

### Language data

There are many different ways of investigating language change, for example considering changes to lexicon, morphology, phonology, or syntax (Bowern and Evans, 2014). Here we consider one particular form of language evolution, the gain, and loss of word variants from basic vocabulary, as it allows us to make comparable measures of rate of language change across different languages (Bromham et al., 2015a). Basic vocabulary consists of a common set of concepts found in all languages, such as “hand,” “mother,” or “water,” for which the common word forms have been recorded in different languages—sometimes referred to as a Swadesh list (Swadesh, 1955).

We used published databases of the different words (lexemes) used for a defined set of basic concepts (semantic categories). Using curated databases ensures that word forms are recorded in a comparable format for the different languages within a family. Each of the databases identifies cognate sets: forms which exhibit some systematic degree of similarity and are identified as derived from a common ancestor (Durie and Ross, 1996; Bowern and Evans, 2014). For example, the semantic category “tree” is represented by different words in different Indo-European languages. In some languages, the words for “tree or wood” reflect the same homologous cognate class derived from the common proto-Indo-European ^*^deru-o- (Derksen, 2008), including 
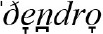
 (Greek), 
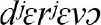
 (Russian), and English *tree* (via Old English, *trēow*). In contrast, the Italic languages have adopted a new lexeme reflected in forms like Latin *arbor*, French *arbre*, Italian *albero* and Spanish á*rbol*. Homologous forms are not just look-alikes but are identified using the linguistic comparative method to determine systematic sound correspondences and phonological innovations (Paul, 1880; Bloomfield, 1933; Durie and Ross, 1996; Bowern and Evans, 2014). We can use these patterns of homology to identify the presence of words shared by descent, the loss of shared cognates from related languages, and also to identify cases of gain of new words that have not been inherited from a common ancestor.

For the Austronesian languages we used the Austronesian Basic Vocabulary Database (ABVD, Greenhill et al., 2008) which contains wordlists for 210 semantic categories from 1,278 languages. For the Indo-European languages, we used the Indo-European Lexical Cognacy Database (IELex, Bouckaert et al., 2012), which contains wordlists for 225 semantic categories from 163 languages. Basic vocabulary for 100 words from 409 Bantu languages were provided by Grollemund et al. (2015) in a phylogenetic dataset that records a single variant per semantic category for each language. The wordlists in these three databases are not identical as they have been modified to contain region specific words, but the lists do overlap substantially as they are based on standard Swadesh lists (Swadesh, 1952).

### Language pairs

To control for relatedness between languages and avoid Galton's problem, we use a simple and robust method of selecting phylogenetically independent sister pairs. Sister pairs are each other's closest relatives on a phylogeny that form a pair of tips connected by their most recent common ancestor. This means that any difference between the two sister languages has arisen since that last common ancestor, and changes in one language are independent of changes in its sister language. Therefore we can ask questions such as: when two languages evolve from a common stock, does the language with the smaller population acquire new words at a greater or lesser rate than the larger language? If we select sister pairs that are each other's closest relatives, such that they share a more recent common ancestor with each other than either shares with any other language in the analysis, then the pairs are said to be phylogenetically independent (Felsenstein, 1985; Harvey and Pagel, 1991), because any differences between the pair has evolved since their common ancestor, and is not a result of their shared inheritance. Selecting phylogenetically independent sister pairs is like running an experiment over and over again, taking one language, splitting it in two, and seeing which one evolves faster (Bromham, 2016). Given sufficient independent comparisons we can use statistical analysis to look for consistent patterns between the features of languages and their rate of change, by comparing them to their sister languages.

The sister pairs approach has advantages over whole tree phylogenetic methods that use every branch in a phylogeny as a datapoint in an analysis. Using only the tips of the phylogeny avoids the need to infer ancestral states at increasing depths down the phylogeny in order to correlate past states with rates of change inferred from the internal branches of the tree. Using only tip branches also avoids the problem of non-independence between ancestor and descendant lineages within the phylogeny, as each branch is likely to be more similar in many traits to its immediate neighbors than it is to other more distantly related branches.

Phylogenetically independent pairs of languages were chosen from published phylogenies and checked for consistency with language taxonomy based on linguistic comparative data. We did not include creoles as they are hybrid languages with a high degree of borrowing and may have different patterns of change to other related languages (Thomason and Kaufman, 1988; Blasi et al., 2017). We did not include extinct or ancient languages, as their lexical documentation may not be as complete as for extant languages, and their speaker population sizes may also be less well established. We included only well-attested sister pairs in our analysis. We began by selecting sister pairs from the published phylogenies (Gray et al., 2009; Bouckaert et al., 2012; Grollemund et al., 2015; Hammarström et al., 2016), then checked the relationship between pairs in the Ethnologue (Lewis et al., 2015). We discarded any pairs where the classification in the Ethnologue was at odds with pairs identified from the phylogeny. We also used phylogenetic support measures from published phylogenies as a guide to selecting well-attested sister pairs, rejecting any pairs with less than 80% posterior probability in the published phylogeny.

Contemporary speaker population size was obtained from the Ethnologue (Lewis et al., 2015) using the *in area* speaker population where given, rather than the total global number of speakers. Languages with insufficient linguistic, temporal or population data were excluded. Thus, this is not an exhaustive list of all sister languages for these language families, but a conservative selection which fits all relevant criteria for this study. This selection process resulted in 81 pairs of Austronesian languages (Table 1), 14 pairs of Indo-European languages (Table 2), and 58 pairs of Bantu languages (Table 3).

**Table 1:** Sister pairs of languages from the Austronesian language family, showing the taxon label, the ISO-639-3 language identification code, the number of gains, losses, and total changes, population size, and branch-length.

**Pair**	**Taxon**	**ISO-639-3**	**Gain**	**Loss**	**Total**	**Population**	**Branch length**
1	Agta	agt	50	32	82	780	138.91
	Gaddang	gad	54	34	88	30,000	
2	AmbaiYapen	amk	112	36	148	10,100	777.07
	WindesiWandamen	wad	117	12	129	5,000	
3	AmbrymSouthEast	tvk	74	45	119	3,700	0.07
	PaameseSouth	pma	51	31	82	6,000	
4	Anakalang	akg	12	23	35	16,000	828.02
	Wanukaka	wnk	23	36	59	10,000	
5	Aputai	apx	14	16	30	150	111.68
	Perai	wet	12	14	26	280	
6	As	asz	86	26	112	230	1905.88
	BigaMisool	xmt	85	25	110	1,250	
7	Atoni	aoz	124	46	170	700,000	1224.40
	RotiTermanu_D	twu	97	18	115	30,000	
8	AttaPamplona	att	26	18	44	1,000	0.00
	Ibanag	ibg	34	28	62	500,000	
9	Avava	tmb	57	67	124	700	552.95
	Neveei	vnm	44	42	86	500	
10	Bali	ban	106	58	164	3,330,000	1897.90
	Sasak	sas	73	59	132	2,100,000	
11	Baree	pmf	80	36	116	137,000	9.22
	Mori	xmz	104	48	152	14,000	
12	Belait	beg	72	34	106	1,000	1107.19
	BerawanLongTerawan	zbw	85	43	128	1,000	
13	Bintulu	bny	70	38	108	4,200	2335.48
	MelanauMukah	mel	68	40	108	113,000	
14	Bobot	bty	47	21	68	4,500	971.12
	Bonfia	bnf	50	17	67	1,000	
15	Bonerate	bna	27	13	40	9,500	0.00
	Popalia	bhq	27	12	39	130,000	
16	BontokGuinaang	bnc	56	34	90	40,700	0.00
	KankanayNorthern	xnn	37	33	70	70,000	
17	BugineseSoppeng_D	bug	80	50	130	5,000,000	2102.00
	TaeSToraja	rob	58	41	99	340,000	
18	Bugotu	bgt	107	51	158	4,050	0.20
	Nggela	nlg	72	30	102	11,900	
19	Bukat	bvk	100	47	147	400	1102.31
	Lahanan	lhn	71	25	96	350	
20	Buli	bzq	114	19	133	2,520	1578.20
	Giman	gzn	152	41	193	2,900	
21	BuruNamroleBay	mhs	110	38	148	33,000	2158.07
	Soboyo	tlv	121	53	174	4,520	
22	Bwaidoga	bwd	52	14	66	6,500	4.10
	Diodio	ddi	57	29	86	2,180	
23	Cebuano	ceb	31	44	75	15,800,000	553.03
	Surigaonon	sgd	70	42	112	400,000	
24	ChekeHolo	mrn	94	61	155	10,800	313.81
	KilokakaYsabel	jaj	34	23	57	10	
25	Dai	dij	52	18	70	820	0.01
	NorthBabar	bcd	50	17	67	1,000	
26	Dehu	dhv	190	22	212	13,000	1722.11
	Nengone	nen	185	30	215	8,720	
27	Dobuan	dob	73	38	111	10,000	667.39
	Molima	mox	81	35	116	4,010	
28	Emae	mmw	4	23	27	400	0.00
	UveaWest	uve	2	24	26	2,200	
29	Gapapaiwa	pwg	76	18	94	3,000	756.15
	Ubir	ubr	101	44	145	2,560	
30	Geser	ges	63	23	86	36,500	476.20
	Watubela	wah	71	36	107	4,000	
31	GhariGuadalcanal	gri	39	31	70	12,100	0.01
	Tolo	tlr	33	33	66	12,500	
32	GorontaloHulondalo	gor	96	50	146	1,000,000	0.12
	Kaidipang	kzp	71	22	93	26,600	
33	HituAmbon	htu	64	27	91	16,000	531.14
	Paulohi	plh	73	33	106	50	
34	HoavaNewGeorgia	hoa	61	41	102	460	400.12
	MarovoNewGeorgia	mvo	67	54	121	8,090	
35	Imroing	imr	31	24	55	560	327.51
	TelaMasbuar	tvm	25	16	41	1,050	
36	Inibaloi	ibl	35	33	68	111,000	117.06
	KallahanKayapaProper	kak	22	20	42	15,000	
37	ItnegBinongan	itb	34	40	74	7,500	0.01
	KalingaGuinaangLubuagan_D	knb	29	36	65	30,000	
38	Jawe	jaz	109	24	133	990	0.00
	Nelemwa	nee	118	26	144	1,090	
39	Kalagan	kqe	33	38	71	70,000	0.00
	Mansaka	msk	25	31	56	57,800	
40	Kapampangan	pam	74	41	115	1,900,000	1165.44
	SambalBotolan	sbl	108	56	164	32,900	
41	Kapingamarangi	kpg	4	18	22	3,000	226.89
	Nukuoro	nkr	3	16	19	860	
42	Kedang	ksx	106	37	143	30,000	1219.42
	Lamaholot	slp	93	33	126	180,000	
43	Kemak	kem	65	16	81	72,000	866.01
	Mambai	mgm	80	27	107	131,000	
44	Kerinci	kvr	56	33	89	260,000	188.09
	Minangkabau	min	29	37	66	5,530,000	
45	Komering	kge	74	37	111	470,000	1899.99
	Lampung	ljp	45	29	74	827,000	
46	KoronadalBlaan	bpr	10	11	21	150,000	415.53
	SaranganiBlaan	bps	4	5	9	90,800	
47	Kuanua	ksd	111	31	142	61,000	652.24
	LungaLungaMinigir	vmg	83	21	104	600	
48	KwaraaeSolomonIslands	kwf	43	33	76	32,400	197.90
	Toambaita	mlu	47	49	96	12,600	
49	Leipon	lek	42	22	64	650	840.70
	Loniu	los	43	20	63	460	
50	Lenakel	tnl	34	25	59	11,500	0.00
	TannaSouthwest	nwi	26	13	39	4,500	
51	Levei	tlx	62	16	78	1,600	1480.51
	Likum	lib	56	13	69	80	
52	Lou	loj	74	36	110	1,000	2.12
	Nauna	ncn	64	25	89	100	
53	Luangiua	ojv	6	14	20	2,370	189.54
	Sikaiana	sky	3	17	20	730	
54	Maanyan	mhy	74	22	96	150,000	1100.00
	MerinaMalagasy	plt	119	54	173	7,520,000	
55	Manam	mva	94	36	130	7,950	171.10
	Wogeo	woc	87	34	121	1,620	
56	Mangareva	mrv	1	28	29	600	670.85
	Marquesan	mrq	23	33	56	5,400	
57	ManoboIlianenKibudtungan_D	mbi	22	34	56	14,600	125.89
	WBukidnonManobo	mbb	23	31	54	15,000	
58	ManoboKalamansigCotabatoParil_D	mta	47	48	95	30,000	306.23
	ManoboSaranganiKayaponga_D	mbs	33	34	67	58,000	
59	Masiwang	bnf	17	4	21	1,000	0.00
	Werinama	bty	19	7	26	4,500	
60	Matukar	mjk	51	16	67	430	556.52
	Megiar	tbc	49	18	67	40,000	
61	Modang	mxd	90	24	114	15,300	339.52
	PunanKelai	sge	83	21	104	2,000	
62	Mokilese	mkj	15	9	24	1,500	1232.98
	Ponapean	pon	34	29	63	31,350	
63	Mortlockese	mrl	2	6	8	5,900	156.44
	Satawalese	stw	1	7	8	460	
64	Mota	mtt	87	33	120	900	933.62
	Mwotlap	mlv	68	42	110	1,800	
65	Naman	lzl	52	42	94	15	415.28
	Tape	mrs	70	74	144	15	
66	Ngadha	nxg	77	26	103	60,000	162.76
	Soa	ssq	73	36	109	10,000	
67	NgaiborSAru	txn	100	19	119	7,910	1319.07
	UjirNAru	udj	89	8	97	1,030	
68	Nguna	llp	57	24	81	9,500	2179.01
	SouthEfate	erk	62	39	101	6,000	
69	Niue	niu	12	52	64	2,030	0.00
	UveaEast	wls	7	25	32	9,620	
70	PeteraraMaewo	mwo	45	44	89	1,400	1667.29
	Raga	lml	47	38	85	6,500	
71	Rurutuan	aut	38	19	57	3,000	31.67
	TahitianModern	tah	27	33	60	68,260	
72	Saliba	sbe	82	29	111	2,500	0.00
	Suau	swp	48	24	72	6,800	
73	SangilSaraganiIslands	snl	42	41	83	15,000	497.32
	SangirTabukang_D	sxn	20	19	39	255,000	
74	Seimat	ssg	98	39	137	1,000	2128.97
	Wuvulu	wuv	100	35	135	1,000	
75	Serili	sve	27	14	41	330	480.25
	SouthEastBabar	vbb	21	10	31	4,460	
76	SubanonSiocon	suc	47	17	64	125,000	415.21
	SubanunSindangan	syb	50	23	73	140,000	
77	SyeErromangan	erg	53	15	68	1,900	1828.80
	Ura	uur	61	28	89	6	
78	Taiof	sps	88	39	127	1,400	26.49
	Teop	tio	129	40	169	5,000	
79	Tigak	tgc	63	39	102	6,000	558.85
	TungagTungakLavongai	lcm	123	22	145	12,000	
80	Tokelau	tkl	12	45	57	1,410	1428.51
	Tuvalu	tvl	4	21	25	10,700	
81	VaghuaChoiseul	tva	63	35	98	1,960	0.01
	Varisi	vrs	40	20	60	5,160	

**Table 2:** Sister pairs of languages from the Indo-European language family, showing the taxon label, the ISO-639-3 language identification code, the number of gains, losses, and total changes, population size, and branch-length.

**Pair**	**Taxon**	**ISO-639-3**	**Gain**	**Loss**	**Total**	**Population**	**Branch length**
1	Persian_List	pes	16	36	52	45,000,000	788.52
	Tadzik	tgk	39	26	65	6,380,000	
2	Romanian_List	ron	41	19	60	19,900,000	727.95
	Vlach	rup	31	44	75	50,000	
3	Sardinian_C	sro	12	22	34	500,000	615.19
	Sardinian_N	src	20	29	49	500,000	
4	Ladin	lld	13	18	31	31,000	649.30
	Romansh	roh	20	33	53	40,000	
5	French	fra	2	11	13	60,000,000	522.68
	Walloon	wln	20	26	46	600,000	
6	Portuguese_ST	por	36	24	60	10,000,000	337.65
	Spanish	spa	19	36	55	38,400,000	
7	Irish_A	gle	40	25	65	138,000	563.10
	Scots_Gaelic	gla	47	25	72	58,700	
8	Dutch_List	nld	7	17	24	15,700,000	208.55
	Flemish	vls	5	22	27	1,070,000	
9	German_ST	deu	7	14	21	69,800,000	641.05
	Luxembourgish	ltz	17	30	47	266,000	
10	Faroese	fao	9	14	23	66,000	777.56
	Icelandic_ST	isl	7	27	34	230,000	
11	Bulgarian	bul	19	44	63	7,020,000	712.58
	Macedonian	mkd	32	14	46	1,340,000	
12	Lusatian_L	dsb	4	8	12	6,670	54.80
	Lusatian_U	hsb	1	5	6	13,300	
13	Byelorussian	bel	15	45	60	2,220,000	535.34
	Ukrainian	ukr	42	26	68	32,000,000	
14	Latvian	lav	68	46	114	1,470,000	1359.36
	Lithuanian_ST	lit	61	40	101	2,800,000	

**Table 3:** Sister pairs of languages from the Bantu language sub-family, showing the taxon label, the ISO-639-3 language identification code, the number of gains, losses, and total changes, population size, and branch-length.

**Pair**	**Taxon**	**ISO-639-3**	**Gain**	**Loss**	**Total**	**Population**	**Time**
1	A15C_Akossi	bss	1	7	8	100,000	479.35
	A15C_Mkaa	bqz	3	9	12	30,000	
2	A24_Duala	dua	0	11	11	87,700	684.16
	A27_Malimba	mzd	5	16	21	2,230	
3	A32C_Batanga	bnm	0	9	9	9,000	572.43
	A34_Benga	bng	2	11	13	3,900	
4	A41_Barombi-Kang	bbi	3	11	14	3,000	526.00
	A42_Abo	abb	0	8	8	12,000	
5	A44_Tunen	tvu	8	23	31	35,300	1226.99
	A46_Nomaande	lem	12	27	39	6,000	
6	A62B_Mmala	mmu	0	1	1	8,000	317.48
	A62C_Libie	ekm	4	5	9	6,400	
7	A841_Badwe	ozm	1	3	4	40,000	149.27
	A84_Njem	njy	0	2	2	4,400	
8	A91_Kwakum	kwu	12	25	37	10,000	1193.38
	A93_Kako	kkj	8	21	29	100,000	
9	B201_Ndasa	nda	0	2	2	4,530	182.77
	B24_Wumbvu	wum	2	4	6	18,300	
10	B252_Mahongwe	mhb	1	10	11	8,000	433.10
	B25_Kota	koq	2	11	13	25,000	
11	B301_Viya	gev	4	23	27	50	1263.89
	B305_Vove	buw	1	20	21	4,000	
12	B304_Pinzi	pic	1	7	8	1,000	251.89
	B32_Kande	kbs	2	8	10	500	
13	B52_Nzebi	nzb	1	7	8	120,000	350.62
	B53_Tsaangi_Poungi	tsa	2	8	10	13,600	
14	Bamun_Grassfields	bax	7	7	14	420,000	536.22
	Mungaka_Grassfields	mhk	6	6	12	50,100	
15	C142_Mondongo	bui	1	8	9	4,000	313.36
	C412_Libobi	bmg	2	9	11	20,000	
16	C37_Ebudza	bja	8	16	24	226,000	1116.06
	C42_Ebwela	bwl	12	20	32	8,400	
17	C71_Tetela	tll	6	19	25	750,000	930.34
	C76_Ombo	oml	4	17	21	8,400	
18	C83_Bushong	buf	0	10	10	155,000	751.44
	C85_Wongo	won	2	12	14	12,700	
19	D201_Liko	lik	10	31	41	60,000	1176.87
	D21_Baali	bcp	11	32	43	42,000	
20	D305_Nyanga-li	nyc	4	4	8	48,000	583.87
	D43_Nyanga	nyj	4	4	8	150,000	
21	D333_Ndaaka	ndk	3	8	11	25,000	467.68
	D334_Mbo	zmw	5	10	15	11,000	
22	E72a_Giryama	nyf	2	14	16	944,000	600.19
	E73_Digo	dig	5	17	22	313,000	
23	E74a_Dawida	dav	8	23	31	274,000	1081.89
	G39_Saghala	tga	10	25	35	79,000	
24	F12_Bende	bdp	10	29	39	27,000	1126.01
	F23_Sumbwa	suw	1	20	21	191,000	
25	F24_Kimbu	kiv	4	18	22	78,000	762.10
	F31_Nyiramba	nim	7	21	28	455,000	
26	G11_Gogo	gog	2	26	28	1,440,000	813.11
	G12_Kagulu	kki	1	25	26	241,000	
27	G23_Sambaa	ksb	3	14	17	664,000	363.63
	G24_Bondei	bou	2	13	15	50,000	
28	G35_Luguru	ruf	6	21	27	692,000	469.00
	G36_Kami	kcu	1	16	17	16,400	
29	G44D_Maore	swb	4	6	10	92,800	262.27
	G44b_Ndzwani	wni	1	3	4	264,000	
30	G61_Sangu	sbp	1	20	21	75,000	611.71
	G66_Wanji	wbi	6	25	31	28,000	
31	G62_Hehe	heh	3	13	16	805,000	491.97
	G63_Bena	bez	6	16	22	670,000	
32	H16a_Kisikongo_2013	kwy	1	12	13	537,000	695.08
	H16a_Kisolongo_DRC_2012	kng	2	13	15	3,000,000	
33	JD64_Shubi	suj	0	5	5	153,000	288.52
	JD65_Hangaza	han	2	7	9	150,000	
34	JD66_Kiha	haq	3	11	14	990,000	483.82
	JD67_Kivinza	vin	2	10	12	10,000	
35	JE11_Runyoro	nyo	3	10	13	667,000	358.91
	JE12_Rutooro	ttj	5	12	17	488,000	
36	JE13_Runyankore	nyn	0	6	6	2,330,000	342.41
	JE14_Rukiga	cgg	3	9	12	1,580,000	
37	JE21_Runyambo	now	1	9	10	400,000	404.52
	JE22_Haya	hay	1	9	10	1,300,000	
38	JE25_Jita	jit	5	10	15	205,000	494.21
	JE25_Kilegi	reg	3	8	11	86,000	
39	JE31_Lumasaaba	myx	6	9	15	1,120,000	544.30
	JE31c_Bukusu	bxk	9	12	21	1,433,000	
40	K332_Rumanyo	diu	2	11	13	10,200	783.09
	K33_Kwangali	kwn	3	12	15	73,100	
41	Kom_Grassfields	bkm	1	2	3	233,000	407.67
	Oku_Grassfields	oku	5	6	11	87,000	
42	L31a_Luba-Kasai	lua	2	12	14	6,300,000	1144.85
	L32_Kanyok	kny	6	16	22	200,000	
43	L35_Sanga	sng	1	8	9	431,000	570.02
	L41_Kaonde	kqn	0	7	7	206,000	
44	M11_Pimbwe	piw	1	7	8	29,000	429.35
	M12_Lungwa	rnw	1	7	8	18,000	
45	M21_Ndali	ndh	7	23	30	150,000	734.25
	M31_Nyakyusa	nyy	7	23	30	805,000	
46	M21_Wanda	wbh	1	4	5	24,000	203.84
	M22_Namwanga	mwn	0	3	3	140,000	
47	M24_Malila	mgq	2	19	21	65,000	414.18
	M25_Safwa	sbk	4	21	25	158,000	
48	M52_Lala	leb	1	4	5	353,000	293.42
	M54_Lamba	lam	1	4	5	201,000	
49	M61_Lenje	leh	2	7	9	128,000	643.12
	M62_Soli	sby	9	14	23	34,100	
50	Moghamo_Grassfields	mgo	9	9	18	183,000	715.68
	Njen_Grassfields	njj	6	6	12	1,800	
51	N11_Manda	mgs	1	18	19	22,000	671.25
	N12_Ngoni	ngo	3	20	23	170,000	
52	N13_Matengo	mgv	5	16	21	150,000	545.66
	N14_Mpoto	mpa	0	11	11	80,000	
53	N31_Chewa	nya	6	18	24	7,000,000	755.02
	N42_Kunda	kdn	1	13	14	145,000	
54	P21_Yao	yao	10	16	26	2,200,000	598.01
	P22_Mwera	mwe	5	11	16	469,000	
55	P31G_Ikorovere	mgh	6	6	12	963,000	390.78
	P31_Emakhua	vmw	2	2	4	3,090,000	
56	S11_Shona	sna	4	13	17	10,700,000	858.44
	S16_Kalanga	kck	6	15	21	700,000	
57	S311_Shekgalagari	xkv	8	14	22	40,000	557.18
	S31_Tswana	tsn	5	11	16	1,070,000	
58	S51_Tshwa	tsc	2	6	8	1,160,000	276.76
	S53_Tsonga	tso	1	5	6	2,280,000	

*Language identification codes following Guthrie's scheme are prepended to the taxon label*.

Language pairs that have a shorter period of divergence will have larger uncertainty in the estimates of their rates of language change (Welch and Waxman, 2008; Hua et al., 2015), so we use estimated branch lengths between sister languages to correct for this effect. We extracted branch lengths from the published language phylogenies (Gray et al., 2009; Bouckaert et al., 2012; Grollemund et al., 2015) which are estimated using phylogenetic dating methods from their total datasets combined with historical and archeological information (Tables 1, 3). Because the relative height of the ancestral node of any given pair will be determined not only by the differences between the pair but also by rates of change estimated on the rest of the phylogeny, it should be at least partially independent of the number of gains and losses between members of any given pair. Branch lengths were only used for the Welch & Waxman analysis (see below).

### Comparing rates of language change

We use comparisons of words from basic vocabulary between pairs of closely-related languages to identify instances of gain and loss of words. We identified patterns of word gain and loss by recording instances where a cognate form within a given semantic category was present in one language in a sister pair but not found in its sister language (Bromham et al., 2015a). A cognate class is a set of words identified as derived from a common ancestor, and therefore the presence of a cognate class in one language of a pair, and in other languages within the family, implies the presence of that cognate class in the common ancestral language of the pair. This method differs from approaches where the net dissimilarity between lists of terms is compared (Wichmann and Holman, 2009). Instead we use only those words that show a pattern of occurrence that is informative for determining differences in rates of gain and loss of words (Bromham et al., 2015a).

**Figure 2:**
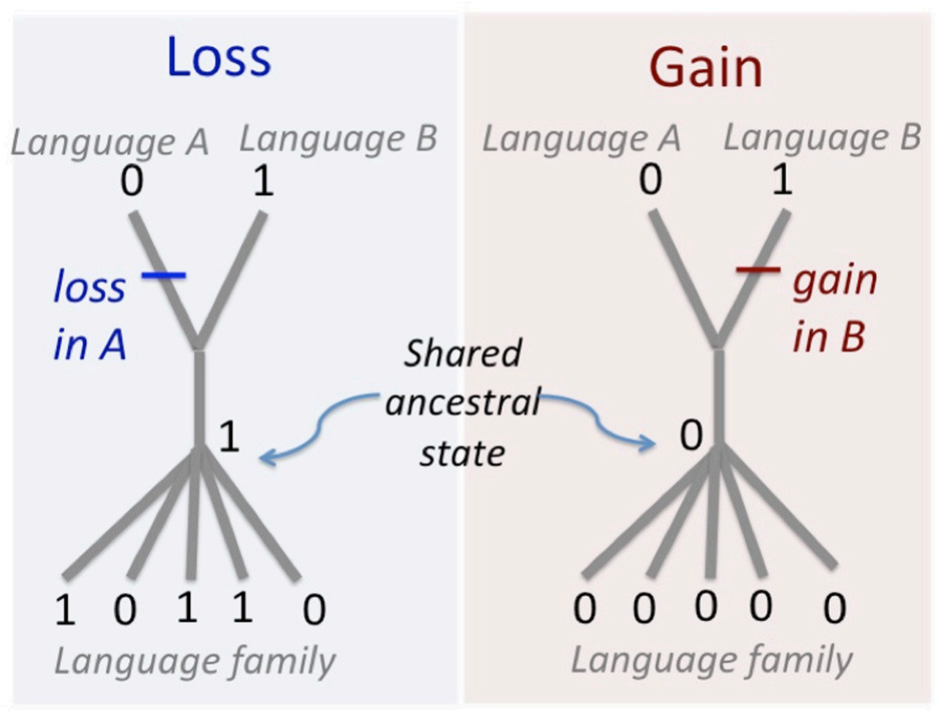
Method for determining word gains and losses. If a cognate form is found in one member of a sister pair and in another language in the family, it must have been lost from the other sister language. A lexeme that has no cognates in any other language in the family, including its sister language, is considered to have been gained since they split from their shared common ancestor.

If a word form found in one sister language has a cognate in other languages in the language family, then it is likely to have been inherited from the common ancestor. This implies that the absence of that cognate form in the other sister language must be due to its loss after divergence from the common ancestor of the pair (Figure 2). If one of the sister languages has a unique word form that has no recognized cognates in any other language in the family, then it presumably represents a gain of a new word since it split from its sister language. Therefore we can identify instances of word gain and loss in both members of a related pair of languages. Any such changes that have occurred in one sister pair of languages can be considered to have happened independently from changes in other sister pair of languages, so these comparisons can be treated as statistically independent data points (Bromham et al., 2015a).

Our analysis only includes cognate classes showing rates-informative patterns that allow us to localize a word gain or loss to only one member of a sister pair (Figure 2). There are two rates-informative patterns. Presence of a cognate class in one member of the pair but not the other indicates a loss of the shared ancestral cognate form from one sister language after divergence from the common ancestor. Presence of a novel form in one member of the pair that has no known cognates in any other member of the language family indicates the gain of a new word in one sister language after divergence from the common ancestor. We did not consider cognate forms that are present in both members of a sister pair because they have both inherited those forms from their common ancestor, and neither has lost that cognate, so those cognates are non-informative for rates of gain and loss. Similarly, we did not count any cognate class that is absent from both members of a sister pair, on the assumption that it was not present in their common ancestor.

We do not include any identified loan words in the analysis, so any cognate terms shared by two languages should be present in the language due to inheritance from a common ancestor, rather than borrowing (horizontal transfer) from another language. The addition of a new word does not necessarily involve the loss of an existing word as languages can have multiple lexemes for one category, therefore each recorded gain, or loss of a lexeme was counted as a separate event, regardless of semantic category. Any lexemes that were recorded as “doubtful” or “exclude” in the databases were excluded from our analysis. Any semantic categories that did not contain entries for both languages in the pair were also excluded as we are unable to ascertain if this absence is a true absence or simply missing data.

This counting procedure will in some cases count semantic shifts as a change (e.g., Danish *træ* “tree” is cognate with proto-Indo-European ^*^dóru but has shifted to also mean “wood”). Due to the nature of these datasets (cognate classes coded *within* a limited number of semantic categories), we cannot quantify semantic shift, which may include gain, or loss of meaning from unrecorded semantic categories. Cognates that change meaning and undergo semantic shifts into a new category in the word list might appear as the gain of a new cognate into the recipient semantic category. If there is a subsequent change of meaning away from the original semantic category, then we would count this as loss of a cognate from the original semantic category. While this represents a somewhat different kind of change from the origin, replacement and loss of lexical items, it is still indicative of language change. In this way, we may include changes in both form and meaning. One of the ways that the population size hypothesis might affect language change is through altering semantics.

The total number of gains, losses, and non-informative results were counted for all available semantic categories for each pair of languages. The raw counts were standardized by the total number of comparisons made between the pairs (gains + losses + non informative + excluded) to allow for comparisons to be made between languages. We have developed a Python package, *RateCounter* (https://github.com/SimonGreenhill/RateCounter), to extract this rate information from common phylogenetic file formats.

### Statistical analysis

We applied two statistical analyses to test for any consistent relationship between population size and rates of word gain and loss. One analysis is Poisson regression (Bromham et al., 2015b; Hua et al., 2015), which assumes that gain and loss counts follow a Poisson process, and rates of word gain and loss are linear functions of population size on a log-log scale (which confines rates to positive values). The regression coefficient between population size and rate of word gain and loss was estimated by accounting for the phylogenetic structure of the data and using a model with stable population size, origination of new language by fission, and negligible founder effect—the simplest population model tests from a previous study (Bromham et al., 2015a). We also tested an alternative model that incorporates population growth, to reflect recent population expansion, however this model provided a poor fit to the data and would not converge for most datasets. Therefore we applied the simplest model because it has the least number of parameters and assumptions and does not require divergence dates. To assess the model fit, we used likelihood ratio tests to compare each model to null models which assume no effect of population size on rates of language evolution. The effect size was calculated as the pseudo *R*^2^ measures for the Poisson regression (Table 1).

In addition, we performed an analysis that first uses the Welch & Waxman test to remove pairs where the divergence between the sister languages is too recent to obtain reliable measures of rates of word gain and loss (Welch and Waxman, 2008). This is done by progressively removing pairs until there is no negative relationship between the absolute value of the standardized difference in the counts of gains and losses between sister languages and the square root of divergence time (Welch and Waxman, 2008), here represented by branch length from the published phylogeny (Tables 1–3). This analysis asks whether the difference in population size between each pair predicts the difference in the gain and loss rate, while accounting for the differences in divergence times between the pairs. So the difference in the gain and loss rate needs to be standardized by divergence times. Since the quantity of data for each language pair may vary, we also need to standardize the differences in the gain and loss rate by the amount of available data. We calculate the standardized difference as the difference in the counts of gains and losses between sister languages divided by their average counts of gains and losses and by the square root of branch length (following Bromham et al., 2015a). We removed any pairs for which the standardized difference was not a reliable estimate of difference in gains or losses rate, for example due to too recent a divergence or insufficient differences between the languages. following the procedure of Welch and Waxman (2008). After removing pairs with unreliable estimates, the analysis then applies least squares regression of the standardized differences between the remaining sister language pairs against their differences in log-transformed population sizes divided by the square root of branch length (Bromham et al., 2015a).

## Results

**Figure 3:**
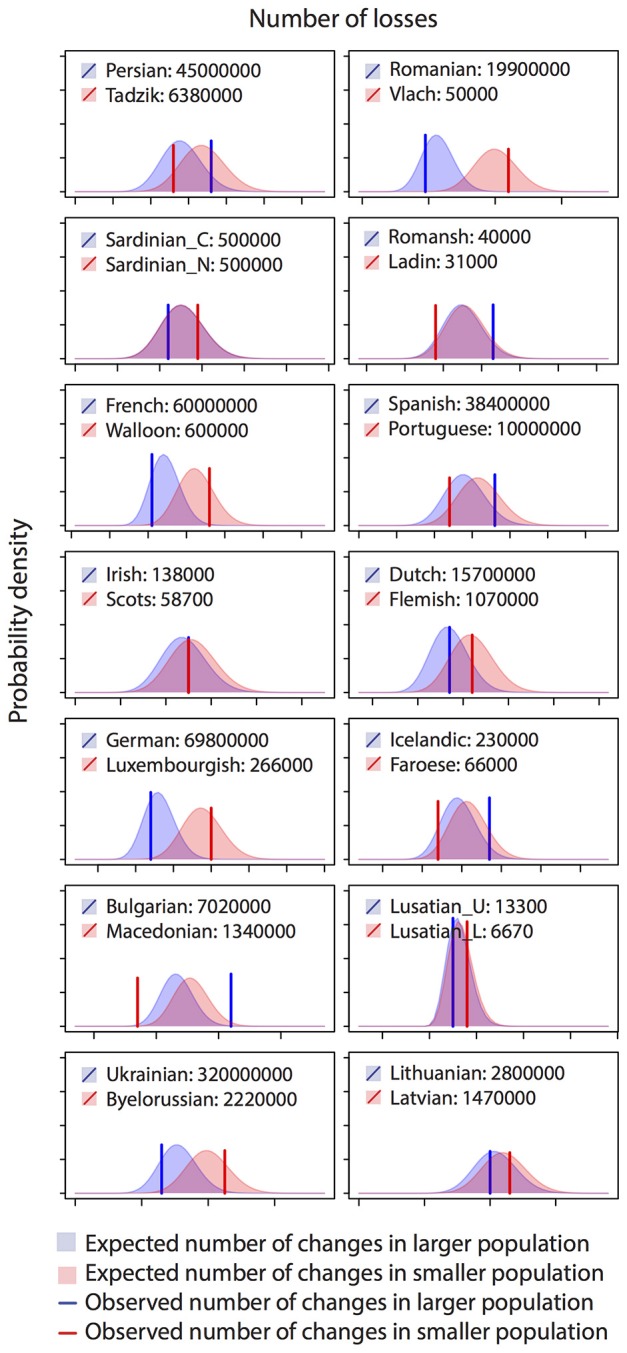
Histograms of observed and expected numbers of word losses in 14 Indo-European language pairs. Plotted distributions show the expected probability of having a certain number of losses for each language, by fitting Poisson regression to all datapoints. Vertical lines show the observed numbers of losses in each language. The language with the larger speaker population size is colored blue while the language with smaller population size is colored red. The analysis reveals a pattern of a smaller population having a faster rate of word loss, with blue line left to red line particularly when difference in population size is large.

**Figure 4:**
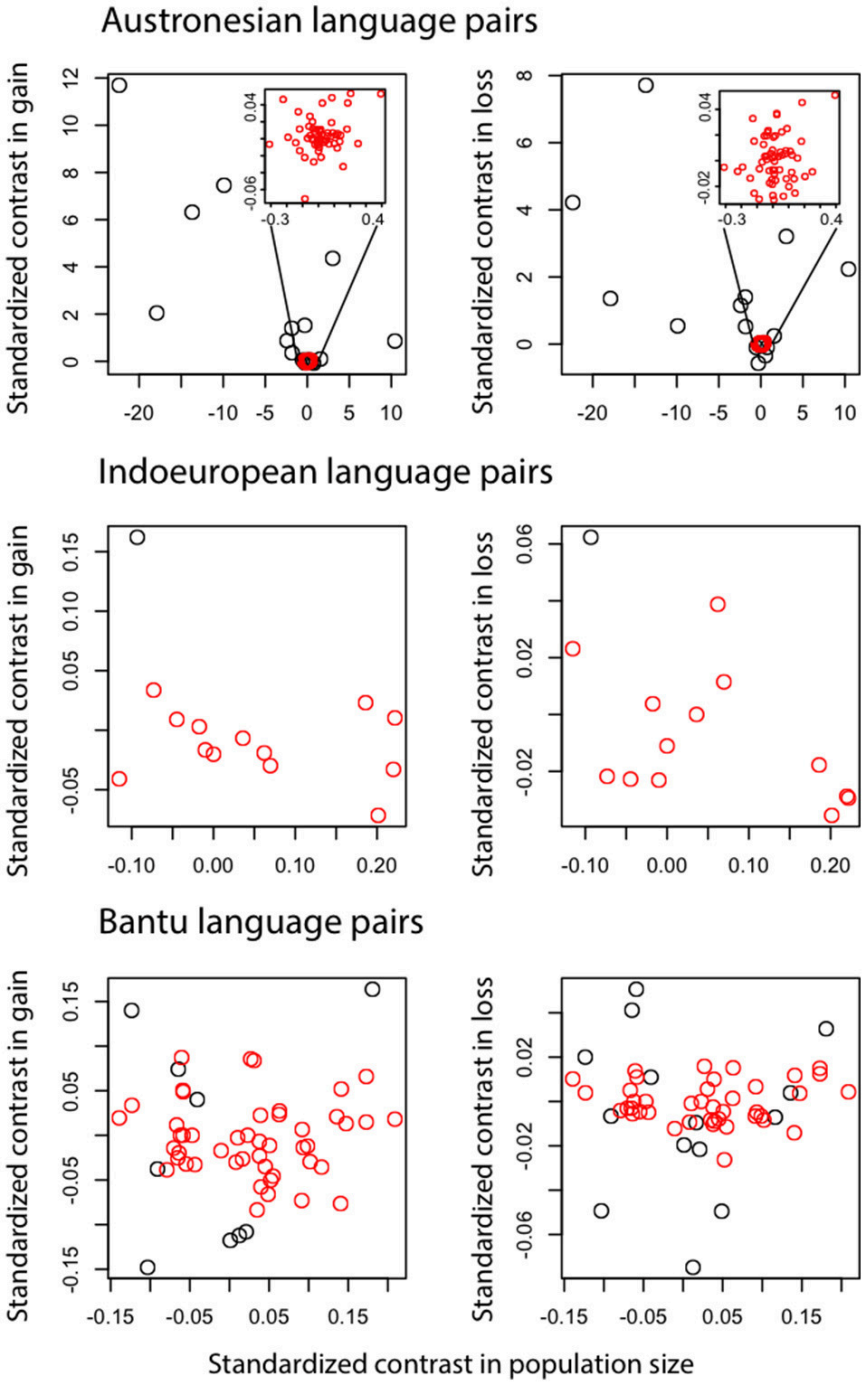
Contrasts in the number of word gains and word losses against contrasts in population size. Each data point represents a language pair, for Austronesian and Indo-European language families and the Bantu subfamily of the Niger-Congo language family. Red data points are language pairs that have reliable estimates for word gain and loss rates according to Welch & Waxman test.

The Poisson regression of population size and rates of change in the Indo-European language family (14 pairs) suggests that languages with smaller speaker population sizes had significantly higher rates of word loss (Table 4, Figure 3). Least squares regression also suggests a significant negative relationship between contrasts in population size and contrasts in the rate of word loss (coefficient = −0.13, *P* = 0.05, *R*^2^ = 0.22). However, this result is no longer significant when a single shallow pair, Upper and Lower Sorbian (Lusatian_U and Lusatian_L) are removed following the Welch & Waxman test (Table 5, Figure 4).

**Table 4:** Results of Poisson regression on Population size and rate of language change in pairs of Austronesian, Indo-European languages, and Bantu languages.

	**N**	**Mean**	**SE**	**Statistic**	***P*-value**	***R*^2^**
**AUSTRONESIAN**						
Gain	81	0.000	0.017	0.07	0.791	0.000
Loss	81	0.001	0.024	0.13	0.718	0.001
**INDO-EUROPEAN**						
Gain	14	−0.042	0.062	2.18	0.140	0.035
Loss	14	−0.095	0.058	**12.82**	**0.000**	0.216
**BANTU**						
Gain	58	−0.000	0.086	0.01	0.911	0.000
Loss	58	−0.000	0.047	0.00	0.951	0.000

*N: number of language pairs; Mean, estimated regression coefficient for the relationship between population size and rates of language change; SE, standard error for the regression coefficient; Statistic, likelihood ratio; P-value, results significant at 0.05 shown in bold; R^2^,pseudo R^2^ for Poisson regression*.

**Table 5:** Results of least squares regression after Welch & Waxman test on Population size and rate of language change in pairs of Austronesian, Indo-European, and Bantu languages.

	**N**	**Mean**	***SE***	**Statistic**	***P*-value**	***R*^2^**
**AUSTRONESIAN LANGUAGES**						
Gain	59	0.041	0.024	3.06	0.086	0.034
Loss	59	0.032	0.021	2.31	0.135	0.022
**INDO-EUROPEAN LANGUAGES**						
Gain	13	−0.047	0.073	0.42	0.532	−0.051
Loss	13	−0.084	0.053	2.52	0.141	0.112
**BANTU LANGUAGES**						
Gain	47	−0.027	0.074	0.13	0.718	−0.019
Loss	41	0.003	0.018	0.02	0.886	−0.025

*N, number of language pairs after removing shallow pairs in regression; Mean, estimated regression coefficient for the relationship between population size and rates of language change; SE, standard error for the regression coefficient; Statistic, F-statistic for least square regression; P-value, results are considered significant at 0.05 level; R^2^, adjusted R^2^ for least square regression*.

We found no evidence of a significant association between rate of word gain and population size in the Indo-European language pairs, nor in gains or losses for the Austronesian and Bantu data (Tables 4, 5, Figure 4). One possible explanation for the observation of a significant relationship between rate of language change and population size only in the Indo-European languages is that we expect this dataset to have relatively higher power to detect differences in rates of change. Although the Indo-European dataset has many fewer pairs than the Austronesian or Bantu datasets, the Indo-European word list contains more cognates per category: that is, there are more synonymous lexemes per word (see Table 6). The test we use to detect rate differences is broadly based on the Tajima test (Tajima, 1993), the power of which is dependent on the number of variable *sites*, which are columns in DNA alignments in which the sequences being compared differ from each other (Bromham et al., 2000). It may be that the more synonyms recorded per lexical category, the more likely we will record a true gain and less likely we will record a false loss (i.e., a synonym is used less frequently in a language but not completely lost). This may be a particular problem for the Bantu dataset which has the fewest synonyms as it was collected following Swadesh's (1952, 1955) approach whereby only the most frequent word was entered for each lexical category. This means that cognates may be retained in lineages even if not recorded, if there are in less frequent usage than a more predominant form. A gain, in this case, may represent the rise in frequency of one cognate over alternatives, therefore may not involve the loss of an alternative form. Given the differences in the nature of the recorded data, we do not know whether the lack of significant relationships for the Bantu and Austronesian data is due to lack of a consistent association between population size and rates of word gain and loss in these language groups, or due to biases in counts of word gain and loss and thus insufficient power to detect rate differences for these datasets.

**Table 6:** Overall statistics for the three cognate datasets showing the language group, source publication, word list size, average number of cognates per language (±standard deviation) and average number of synonyms per lexical entry across languages (±standard deviation).

**Family**	**Data source**	**Word list**	**Cognates**	**Synonyms**
Austronesian	Greenhill et al., 2008	210	198.91 (31.25)	0.95 (0.15)
Indo-European	Bouckaert et al., 2012	207	223.46 (20.95)	1.08 (0.10)
Bantu	Grollemund et al., 2015	100	91.17 (12.29)	0.91 (0.12)

## Discussion

Languages evolve, creating patterns of descent and relatedness reminiscent of biological species. Because of this, tools from evolutionary biology are being increasingly applied to studying language change (Levinson and Gray, 2012; Gavin et al., 2013; Bromham, 2017). However, we cannot assume that the mechanisms underlying change, or the observed patterns and rates of change, will be the same for both languages and biological lineages.

Evolutionary theory makes clear predictions about the relationship between population size and rates and patterns of genetic change. Selection is more efficient in large populations, so deleterious mutations should be removed more effectively, and advantageous mutations should more rapidly go to fixation. However, in smaller populations, random sampling effects can have a comparatively greater impact on the frequency of genetic variants, so that positively selected mutations may be reduced in frequency by chance, and may thus occasionally be lost rather than going to fixation. Conversely, in small populations, slightly deleterious changes may increase in frequency by chance, and may eventually drift to fixation, leading to the loss of other variants at that locus (Charlesworth, 2009; Lanfear et al., 2014).

In contrast, the effects of population size on language evolution are not as straightforward to predict, and many alternative hypotheses have been suggested. Large populations of organisms generate more mutations per generation because there are more genomes in the population that can undergo change. Languages with large speaker populations might be expected to generate more innovations (Kline and Boyd, 2010; Collard et al., 2013), however unlike genetic mutation, the processes that create new language variants are not well understood, and may occur by a wider range of mechanisms. Unlike mutation, which is random with respect to utility, introduction of new language variants can be guided by perceived need, and can be regulated by social convention or top-down rules (see Bromham, 2017). Similarly, rates of language change may show different patterns to genetic change if the process of substitution is by horizontal spread of variants through the population, rather than by inheritance (Reali and Griffiths, 2010). So, unlike adaptive genetic change in biological populations, it is possible that smaller speaker populations might have a greater rate of adoption of innovations because it is easier for new words to diffuse to all speakers and replace all other variants (Nettle, 1999). It is therefore difficult to predict whether smaller or larger speaker populations should have greater rates of language change, whether patterns should be the same or different for both gains and losses of language elements, and whether we expect similar patterns across all language families or more idiosyncratic associations, particular to given language groups.

Our analysis suggests that, as for Polynesian languages, smaller Indo-European languages have greater rates of word loss from basic vocabulary. This result is consistent with the claim that smaller populations are at greater risk of loss of language elements, and other aspects of culture, due to effects of incomplete sampling of variants over generations. However, we note that the relatively small sample size for this dataset complicates the interpretation of this result. Least squares regression after Welch & Waxman test has the same false positive rate but has much less power than Poisson regression when sample size is small (~ten or fewer pairs, Hua et al., 2015). This makes it difficult to interpret the inconsistent results of these two analyses, as they may be due to their difference in the statistical power. Hence, the negative relationship between rates of loss and population size for Indo-European languages would benefit from additional investigation. We do not find evidence for a negative relationship between population size and word loss rates in the Austronesian and Bantu groups. This finding suggests that either these datasets contain too few language variants to have sufficient power to detect rate differences, or that the increased loss rate in small populations is not a universal phenomenon, or that it is a relatively weak force in some language groups and thus may be overwhelmed by other social, linguistic or demographic factors.

One factor that may be playing a role in the uncertainty in our results, and in the wider debate in general, is that measuring speech community size is notoriously difficult. How exactly does one delimit a speech community (Crystal, 2008) and what degree of proficiency in a language is sufficient to be part of the community (Bloomfield, 1933)? This task is made harder as there are few national censuses that collect detailed speaker statistics. Further, speaker population size can change rapidly with many modern world languages (especially the Indo-European languages) experiencing rapid growth over the last few hundred years (Crystal, 2008), while others have experienced catastrophic declines (Bowern, 2010). For the same reasons, the difficulty of obtaining accurate population estimates is also a problem in biology. Furthermore, the relevant parameter for genetic change—the effective population size—is difficult to estimate directly, even when accurate census information is available (Wang et al., 2016). Likewise, there may be an important role played by population and network density—tight-knit networks may inhibit change, while loosely integrated speech communities (regardless of their size), may facilitate change (Granovetter, 1973; Milroy and Milroy, 1992). One way forward here is perhaps to simulate rates of change over a range of population sizes and network topologies (c.f. Reali et al., 2018).

Despite the obvious challenges in obtaining an accurate measure of speaker population size, several previous studies have reported that empirical estimates of population size do correlate with aspects of language change (Hay and Bauer, 2007; Lupyan and Dale, 2010; Bromham et al., 2015a). Therefore, either census population size, as reported in databases such as the Ethnologue, are sufficiently accurate reflections of speaker population size that they are able to reveal significant patterns of language change, or census population size is reflecting some aspect of languages that is connected to change. In either case, the reported relationships with speaker population size invite further investigation.

We can draw two conclusions from these results. Firstly, we provide some evidence that rates of language change can be affected by demographic factors. Even if the effect is not universal, the finding of significant associations between population size and patterns of linguistic change in some languages urges caution for any analysis of language evolution that makes an assumption of uniform rates of change. These results also potentially provide a window on processes of language change in these lineages, providing further impetus to investigate the effect of number of speakers on patterns of language transmission and loss. A more detailed study of language change for a larger number of comparisons might clarify the relationship between population size and word loss rates, particularly within the Indo-European language family.

Secondly, we have shown that the significant patterns of language change identified in a previous study are not a universal phenomenon. Unlike the study of Polynesian languages, we did not find any significant relationships between word gain rate and population size, and the association between loss rates and population size was not evident for all language families analyzed. The lack of universal relationships suggests that it may be difficult to draw general conclusions about the influence of demographic factors on patterns and rates of language change. Many other factors have been proposed to influence rates of language change (Greenhill, 2014) including population density, social structure (Nettle, 1999; Labov, 2007; Ke et al., 2008; Trudgill, 2011), degree of contact, and connectedness with other languages (Matras, 2009; Bowern, 2010), degree of language diffusion within a speech community (Wichmann et al., 2008), degree of bilingualism or multilingualism (Lupyan and Dale, 2010; Bentz and Winter, 2013), language group diversity (Atkinson et al., 2008) and environmental factors such as habitat heterogeneity and latitude (Bowern, 2010; Blust, 2013; Amano et al., 2014). These factors might mediate or overwhelm the effect of speaker population size.

We find no evidence to support the hypothesis that uptake of new words should be faster in small populations, which is based on the assumption that new words can diffuse more efficiently through a smaller speaker population than a larger one (Nettle, 1999). Nor do we find support for the suggestion that large, widespread languages have a tendency to lose linguistic features a greater rate (Lupyan and Dale, 2010). However, this latter hypothesis is predominantly expected to explain loss of complex linguistic morphology (such as case systems), which may be harder for non-native speakers to learn, rather than basic vocabulary studied here which may be comparatively easier for second language learners to acquire (but see Kempe and Brooks, 2018). Further, our results cannot be interpreted as confirmation of previous studies that suggest there is no effect of population size on rates (Wichmann and Holman, 2009). The detection of significant patterns in rates of lexical change with population size variation in the Polynesian and Indo-European languages, but the failure to identify similar patterns in the Bantu and Austronesian data, suggests that patterns of rates may need to be investigated on a case-by-case basis.

The failure to find a consistent association between population size and rate of change for languages means that analogies drawn between biological and linguistic evolution must be carefully considered to make sure that they are appropriate for linguistic evolution (Bowern and Evans, 2014). For example, patterns of human migration can leave similar traces on both genetic and linguistic diversity (Hurles et al., 2003; Hunley et al., 2007, 2008; Longobardi et al., 2015), but even though the patterns are the same, the underlying mechanisms may not be identical. The observation of decreasing phoneme inventories along chains of human migration has been attributed to serial founder effects (Trudgill, 2004; Atkinson, 2011). While founder effect is likely to influence genetic variability, because a small number of colonists cannot carry all of the genetic variation of the parent population, it might not have the same effect on language variants, as the founding population may use all the main variants in basic vocabulary. Similarly, while a correlation between lineage diversity and rate of change has been reported for both genetic and linguistic evolution (Pagel et al., 2006; Atkinson et al., 2008; Lanfear et al., 2010; Bromham et al., 2015a), it may not reflect a shared mechanism: while formation of new languages may drive higher rates of word turnover, speciation itself is unlikely to drive faster mutation rates in molecular evolution. Our results suggest that the population size effects may be another example of a pattern that is superficially similar between linguistic and biological evolution, yet may be driven by different mechanisms.

However, although the processes underlying language change and genetic change may be different, many of the same analytical tools can be used in the study of both biological and language evolution (see Bromham, 2017). This point was well recognized by early promoters of cross-disciplinary dialogue between evolutionary biology and historical linguistics (Morpugo Davies, 1975), such as Charles Darwin, August Schleicher, and Charles Lyell (Lyell, 1863; Schleicher, 1869; Darwin, 1871). For example, Schleicher's analogy between borrowing from a foreign language and biological cross-breeding did not imply the same mechanism for both, yet both have the effect of confounding attempts to represent evolutionary history as a bifurcating phylogeny (List et al., 2014). Yet the same solutions may apply to both processes, regardless of their mechanistic origin, such as representation of relationships as a network rather than a tree. Similarly, the shared problem of phylogenetic non-independence due to shared inheritance applies to both languages and species despite the many differences in mode of evolutionary change. While some solutions may be more readily applied to cross-species analysis, due to the availability of phylogenies for many groups, other solutions can be applied more readily to both languages and species, even in the absence of a phylogeny. We demonstrate here that sister pairs analysis is a viable solution to Galton's problem, and it can be applied using information from widely available language taxonomies.

## Conclusion

Our results show that some of the variation of rates of lexical change in languages can, in some cases, be attributable to differences in speaker population size. Significant correlations between population size and rate of word loss were identified for Indo-European languages, but not for Austronesian and Bantu languages. One possible explanation for the negative relationship between speaker population size and loss rates is that language evolution shares similar mechanisms with genetic evolution, because both show patterns of greater rates of loss of variation in small populations. However, the lack of significant relationships between word gain and loss in two other large language groups—Austronesian and Bantu—warns that we cannot reliably predict variation in rates of linguistic evolution by extrapolation from general principles. By demonstrating that differences can exist in rates of change even between closely related languages, our results caution against assuming uniform rates of change across all languages, and suggest that in some cases the rates of change may be consistently influenced by demographic factors.

## Author contributions

LB, CW, XH, and SG: Conceived the project and wrote the paper; SG, CW, and HS: Collected data; XH: Analyzed data.

### Conflict of interest statement

The authors declare that the research was conducted in the absence of any commercial or financial relationships that could be construed as a potential conflict of interest.

## Footnotes

^1^The current population of Germany is ~82 million speakers, but Lewis et al. (2015) cites a 2012 European Commission report for Standard German which indicates 69.8 million native speakers.
